# On a stochastic order induced by an extension of Panjer’s family of discrete distributions

**DOI:** 10.1007/s00184-021-00822-5

**Published:** 2021-05-13

**Authors:** Aleksandr Beknazaryan, Peter Adamic

**Affiliations:** 1 Department of Applied Mathematics, University of Twente, Enschede, The Netherlands; 2 Department of Mathematics and Computer Science, Laurentian University, Sudbury, Canada

**Keywords:** Discrete distribution, Stochastic order, Recursion, Compound distribution

## Abstract

We factorize probability mass functions of discrete distributions belonging to Panjer’s family and to its certain extensions to define a stochastic order on the space of distributions supported on ${\mathbb {N}}_0$. Main properties of this order are presented. Comparison of some well-known distributions with respect to this order allows to generate new families of distributions that satisfy various recurrrence relations. The recursion formula for the probabilities of corresponding compound distributions for one such family is derived. Applications to various domains of reliability theory are provided.

## Introduction and motivation

Panjer (1981) considered the family of discrete random variables *X* satisfying the relation

1 $$\begin{aligned} p(n)=\frac{an+b}{n}p(n-1), \quad n\ge 1, \end{aligned}$$

where $p(n)=\text {P}(X=n)$, and derived a recursive formula for calculating compound distributions for the case when the corresponding claim number distribution belongs to that family. Being not only a quite important but also a relatively convenient class of distributions to deal with for solving various problems in actuarial science and, in particular, risk theory, Panjer’s family and its certain extensions have subsequently attracted the attention of many researchers. In particular, Sundt and Jewell (1981) have shown that the above family consists of the collection of all (nondegenerate) Binomial, Poisson and Negative Binomial distributions, Willmot (1988) characterized the distributions with $p(0)=0$ that satisfy relation (1) for $n\ge 2$ and Hess et al. (2002) identified the distributions with $p(n)=0$ for $n\le k-1$ that satisfy relation (1) for $n\ge k+1$ (the so called Panjer’s class of order *k*). Also Panjer and Willmot (1982) considered the class of distributions satisfying the recursion

2 $$\begin{aligned} p(n)=\frac{\sum \nolimits _{i=0}^ka_in^i}{\sum \nolimits _{i=0}^kb_in^i}p(n-1), \quad n\ge 1, \end{aligned}$$

for some $k\in {\mathbb {N}}$, and derived recursions for the correponding compound distributions when $k = 1$ and $k = 2$. A recursive algorithm for the compound distribution for arbitrary *k* had been then derived by Hesselager (1994). Extesions of the Panjer’s family to distributions that satisfy recurrence relation of higher orders have also been studied in the literature. In particular, Kitano et al. (2005) considered the generalized Charlier distribution, which satisfies second order recurrence relation

$$\begin{aligned} p(n)=\bigg [a+\frac{b}{n}\bigg ]p(n-1)+\bigg [c+\frac{d}{n}+\frac{e}{n-1}\bigg ]p(n-2), \quad n>1. \end{aligned}$$

A quite extensive collection of other generalizations of Panjer’s family as well as their applications can be found in Sundt and Vernic (2009).

In this work we first consider the family of discrete distributions supported on ${\mathbb {N}}_0={\mathbb {N}}\cup \{0\}$ that satisfy (2). For the sake of simplicity, we assume that the denominator is a monomial $n^m$ and show in the following section that the probability mass function *p*(*n*) can be decomposed into a finite product

3 $$\begin{aligned} p(n)=c\prod _{i}p_i(n), \quad n\ge 0, \end{aligned}$$

where the constant $c>0$ does not depend on *n* and each $p_i$ is a probability mass function of one of four different types of distributions, including the Poisson and the Negative Binomial distributions. Parameters of those distributions are determined by the coefficients that appear in the right-hand side of (2). As the values of probability mass functions are always in between 0 and 1, the above factorization implies that *p*(*n*) is asymptotically smaller than each of $p_i(n)$. This observation leads to the Definition 2.1 of a discrete stochastic order $\preceq$ over the set $\Omega$ of *all* discrete random variables supported on ${\mathbb {N}}_0$ and our main goal is to study the properties of this order.

Stochastic orderings have a modest, yet somewhat punctuated, pedigree in the corpus of statistical literature. A quite extensive collection of such orderings can be found in Shaked and Shanthikumar (2007). Also in Artzner et al. (1999), Artzner et al introduced their now famous four coherent risk measures. However, as stated in Giovagnoli and Wynn (2010), most of the existing literature focuses on real (continuous) univariate or multivariate random variables, and there is less work on stochastic orderings for discrete random variables. In terms of ordering functions in a discrete setting, Kitaev (2005) considers partially ordered generalized patterns, and for discrete random variables, in Giovagnoli and Wynn (2010) use Möbius inversions in the theory as well as the notion of dual cones.

The paper is structured as follows: in Sect. 2 we derive the factorization formula (3) and describe the distributions that appear on the right-hand side of that formula. Based on that decomposition we then define the order $\preceq$ on the whole space of discrete distributions that are supported on ${\mathbb {N}}_0$. To make the definition of this order more intuitive, in Sect. 3 we give two more definitions of $\preceq$ that are equivalent to Definition 2.1. In the following proofs we mainly use the condition given in Proposition 3.1 (i) (though from the statistical point of view the condition given in Proposition 3.1 (ii) may seem to be more interesting). We then show that $\{\Omega , \preceq \}$ is a partially ordered set and that $\preceq$ is uncorrelated with other well-known stochastic orders on $\Omega$. One of the main properties of $\prec$ that distinguishes it from other stochastic orders is its invariance under the permutations of ${\mathbb {N}}_0$ (Proposition 3.6). In particular, this means that $X\prec Y$ does necessarily imply neither ${\mathbb {E}}[X]\le {\mathbb {E}}[Y]$ nor $var[X]\le var[Y].$ Instead, as $\prec$ is determined by the tail behaviour of the distributions, the relation $X\prec Y$ implies $\lim \nolimits _{n\rightarrow +\infty }R_X(n)/R_Y(n)=0,$ where $R_X(n)$ and $R_Y(n)$ are survival functions of *X* and *Y* (Theorem 3.1). However, the converse is not true, that is, the condition $\lim \nolimits _{n\rightarrow +\infty }R_X(n)/R_Y(n)=0$ is in general much weaker than $X\prec Y$ (Examples 3.1 and 3.2). Other characteristics of the partially ordered set $\{\Omega , \preceq \}$, such as the existence and the structure of minimal, maximal and covering elements, are also given in Sect. 3. The relation with respect to $\prec$ of compound distributions for which the corresponding counting distributions are from $\Omega$ is described in Sect. 4. In particular, the conditions are presented under which a compound distribution $S'$ is $\prec$ than a compound distribution $S''$ if and only if either the claim size distribution of $S'$ is $\prec$ than the claim size distribution of $S''$ (Proposition 4.1) or the counting distribution of $S'$ is $\prec$ than the counting distribution of $S''$ (Proposition 4.2). In Sect. 5 we consider some common distributions that can be recursively generated and discuss their relation with respect to $\prec$. This comparison allows to obtain new families of distributions that satisfy various recurrence relations. We then provide a recursive formula for calculating the probabilities of compound distributions whose counting distributions belong to one of those families. Finally, in the last section we present some applications of the obtained results to reliability theory and to Poisson shock models and show that the relation $\prec$ is also applicable while conducting sampling with visibility bias.

## Factorization of probability mass functions

Consider a family of discrete random variables *X* satisfying the relation

4 $$\begin{aligned} p(n)=\frac{P_k(n)}{n^m}p(n-1), \quad n\ge 1, \end{aligned}$$

where $p(n)=\text {P}(X=n)$ and $P_k(x)=\sum \nolimits _{i=0}^ka_ix^i$ is a polynomial of degree $k\ge 0$. Note that when $m=1$ and $k\le 1$ then (4) corresponds to the Panjer’s family of discrete distributions given by (1). We are mainly interested in distributions of the above family that have infinite support ${\mathbb {N}}_0$, which implies that $k\le m$ and $P_k(n)>0$ for all $n\in {\mathbb {N}}$.

Let us first consider the case $m=k$. In this case we have by the ratio test that the series $\sum \nolimits _{n=0}^\infty p(n)$ converges if $|a_k|<1$ and it diverges if $|a_k|>1$. Let us therefore assume that $|a_k|<1$. This also means that for each $M\in {\mathbb {N}},$ the number of zeros of the polynomial $P_k(x)$ in the interval $(M,M+1)$ is even (possibly 0). Hence, we can rewrite the polynomial $P_k(x)$ in the form

5 $$\begin{aligned} P_k(x)=\bigg [\prod \limits _{i_1=1}^{k_1}(ax+b_{i_1})\bigg ]\bigg [\prod \limits _{i_2=1}^{k_2}(a^2x^2+c_{i_2}x+d_{i_2})\bigg ]\bigg [\prod \limits _{i_3=1}^{k_3}(a^2x^2+e_{i_3}x+f_{i_3})\bigg ],\nonumber \\ \end{aligned}$$

where $a=a_k^{1/k},$ $k_1+2k_2+2k_3=k$, the root of each linear function $ax+b_{i_1},$ $i_1=1,..,k_1,$ is less than 1, the (possibly equal) roots of each quadratic function $a^2x^2+c_{i_2}x+d_{i_2}$ lie in the interval $(M_{i_2},M_{i_2}+1)$ for some $M_{i_2}\in {\mathbb {N}},$ $i_2=1,..,k_2,$ and the functions $a^2x^2+e_{i_3}x+f_{i_3},$ $i_3=1,..,k_3,$ have no real roots. We can then rewrite (4) in the form

6 $$\begin{aligned} p(n)= & {} \bigg [\prod \limits _{i_1=1}^{k_1}\frac{ax+b_{i_1}}{x}\bigg ]\bigg [\prod \limits _{i_2=1}^{k_2}\frac{a^2x^2+c_{i_2}x+d_{i_2}}{x^2}\bigg ]\bigg [\prod \limits _{i_3=1}^{k_3}\frac{a^2x^2+e_{i_3}x+f_{i_3}}{x^2}\bigg ]p(n-1),\nonumber \\ n\ge & {} 1, \end{aligned}$$

Let us now describe the distributions which are recursively generated by each of those three types of functions.

It has been shown in Sundt and Jewell (1981) that the only distribution satisfying

7 $$\begin{aligned} p_{i_1}(n)=\frac{an+b_{i_1}}{n}p_{i_1}(n-1), \quad n\ge 1, \end{aligned}$$

with $0<a<1$ is the Negative Binomial distribution with parameters $(a+b_{i_1})/a$ and *a*. More precisely, in this case we have that

8 $$\begin{aligned} p_{i_1}(n)= & {} \frac{an+b_{i_1}}{n}p_{i_1}(n-1)=p_{i_1}(0)\prod \limits _{j=1}^{n} \frac{aj+b_{i_1}}{j}\nonumber \\= & {} p_{i_1}(0)a^n\left( {\begin{array}{c}\frac{a+b_{i_1}}{a}+n-1\\ n\end{array}}\right) , \quad n\ge 0, \end{aligned}$$

where the last equality above follows from the definition of binomial coefficients with real arguments. Note that the condition that the root of $ax+b_{i_1}$ is less than 1 together with $a>0$ implies that $a+b_{i_1}>0$.

Now consider the distribution satisfying

9 $$\begin{aligned} p_{i_2}(n)=\frac{a^2n^2+c_{i_2}n+d_{i_2}}{n^2}p_{i_2}(n-1), \quad n\ge 1, \end{aligned}$$

where the function $a^2x^2+c_{i_2}x+d_{i_2}$ has two real roots, say $x_{i_2}$ and $y_{i_2}$, both of which belong to the interval $(M_{i_2},M_{i_2}+1)$ for some $M_{i_2}\in {\mathbb {N}}$. We then have that

10 $$\begin{aligned} p_{i_2}(n)= & {} \frac{a^2(x_{i_2}-n)(y_{i_2}-n)}{n^2}p_{i_2}(n-1)\nonumber \\= & {} p(0)\prod \limits _{j=1}^{n}\frac{a^2(x_{i_2}-j)(y_{i_2}-j)}{j^2} =p(0)a^{2n}{x_{i_2}-1\atopwithdelims ()n}{y_{i_2}-1\atopwithdelims ()n},\qquad \end{aligned}$$

where the last equality follows from $\Gamma (x)=\Gamma (x-1)(x-1)=\cdots =\Gamma (x-n)\prod _{i=1}^{n}(x-i)$, $x\notin {\mathbb {Z}},$ and from the definition of binomial coefficients with real arguments.

Finally, let us suppose that

11 $$\begin{aligned} p_{i_3}(n)=\frac{a^2n^2+e_{i_3}n+f_{i_3}}{n^2}p_{i_3}(n-1), \quad n\ge 1, \end{aligned}$$

where $a^2x^2+e_{i_3}x+f_{i_3}$ has no real roots. Then $a^2x^2+e_{i_3}x+f_{i_3}$ has 2 complex roots $z_{i_3}$ and ${\overline{z}}_{i_3}$ for some $z_{i_3}\in {\mathbb {C}}{\setminus }{\mathbb {R}}$. Using the properties $z\Gamma (z)=\Gamma (z+1)$ and $\Gamma ({\overline{z}})=\overline{\Gamma (z)}$ of the Gamma function $\Gamma (z)$ we get that

12 $$\begin{aligned} p_{i_3}(n)= & {} \frac{a^2(z_{i_3}-n)({\overline{z}}_{i_3}-n)}{n^2}p_{i_3}(n-1)=p_{i_3}(0) \prod \limits _{j=1}^{n}\frac{a^2(z_{i_3}-j)({\overline{z}}_{i_3}-j)}{j^2}\nonumber \\= & {} p_{i_3}(0) \frac{a^{2n}}{(n!)^2}\frac{|\Gamma (z_{i_3})|^2}{|\Gamma (z_{i_3}-n)|^2}. \end{aligned}$$

Combining (5)–(12) for a given random variable *X* satisfying (4) with $m=k$ we get

13 $$\begin{aligned} \begin{aligned}&p(n)=\frac{P_k(n)}{n^k}p(n-1)=c\bigg [\prod \limits _{i_1=1}^{k_1}p_{i_1}(n)\bigg ] \bigg [\prod \limits _{i_2=1}^{k_2}p_{i_2}(n)\bigg ]\bigg [\prod \limits _{i_3=1}^{k_3}p_{i_3}(n) \bigg ], \quad n\ge 0, \end{aligned}\nonumber \\ \end{aligned}$$

where *c* is some positive constant that does not depend on *n*, $p_{i_1}, p_{i_2}$ and $p_{i_3}$ are probability mass functions of distributions given, respectively, by (8), (10) and (12). In case $m>k$ we can rewrite (4) as

$$\begin{aligned} p(n)=\frac{1}{n^{m-k}}\frac{P_k(n)}{n^k}p(n-1), \quad n\ge 1. \end{aligned}$$

Noting that if $p_0(n)$ denotes the probability mass function of Poisson distribution with parameter 1 then

$$\begin{aligned} p_0(n)=\frac{p_0(n-1)}{n}, \end{aligned}$$

we get that in this case there is a constant number $c>0$ such that

14 $$\begin{aligned} p(n)= & {} \frac{P_k(n)}{n^m}p(n-1)=cp_0(n)^{m-k}\bigg [\prod \limits _{i_1=1}^{k_1}p_{i_1}(n)\bigg ]\bigg [\prod \limits _{i_2=1}^{k_2}p_{i_2}(n)\bigg ]\bigg [\prod \limits _{i_3=1}^{k_3}p_{i_3}(n)\bigg ],\nonumber \\ \quad n\ge 0. \end{aligned}$$

Let us now consider an example that illustrates the above factorization. Suppose *X* follows Poisson distribution with parameter 1/2. Then

$$\begin{aligned} p_X(n)=\frac{e^{-1/2}}{2^nn!}=2e^{1/2}\frac{e^{-1}}{n!}\frac{1}{2^{n+1}}=2e^{1/2}p_Y(n)p_Z(n), \quad n\ge 0, \end{aligned}$$

where $Y\sim$ Poisson(1) and $Z\sim$ Negative Binomial(1, 1/2). Note that the probability mass functions of those variables satisfy recurrence relations $p_X(n)=\frac{1}{2n}p_{X}(n-1),$ $p_Y(n)=\frac{1}{n}p_{Y}(n-1)$ and $p_Z(n)=\frac{1}{2}p_{Z}(n-1),$ and the factor $\frac{1}{2n}$ that appears in the first recursion is a product of the corresponding factors appearing in the two other recursions. Clearly, the values of $p_X(n)$ are then (asymptotically) smaller than the values of $p_Y(n)$ and $p_Z(n)$. In general, as the values of probability mass functions are always in between 0 and 1, then given the decomposition (14) we have that *p*(*n*) is asymptotically smaller than each of the probability mass functions that appear on the right side of (14). Thus, the above decomposition leads to the following definition of a binary relation $\preceq$ over the set $\Omega$ of *all* discrete random variables supported on ${\mathbb {N}}_0$.

### Definition 2.1

Given $X,Y\in \Omega ,$ we say that $X\prec Y$ if there exist a constant number $c\in {\mathbb {R}}_+$ and $Z\in \Omega$ such that

15 $$\begin{aligned} p_X(n)=cp_Y(n)p_Z(n), \quad n\in {\mathbb {N}}_0, \end{aligned}$$

and we say that $X\preceq Y$ if either $X\prec Y$ or $p_X(n)= p_Y(n)$ for all $n\in {\mathbb {N}}_0$.

Note that given (15), *X* can also be considered as a weighted distribution (see, e.g., Patil and Rao (1978) for the definition and properties of weighted distributions). In the following section we show that $\{\Omega , \preceq \}$ is a partially ordered set and derive its main properties.

## Partially ordered set $\{\Omega , \preceq \}$ and properties of $\prec$

Let us first present another 2 equivalent definitons of $\prec$. The definition given in Proposition 3.1 (i) below turns out to be the most convenient one for deriving the subsequent results.

### Proposition 3.1

Given $X,Y\in \Omega$ then $X\prec Y$ if and only if one of the following holds:

(i) $$\sum \limits _{n=0}^\infty \frac{p_X(n)}{p_Y(n)}<\infty $$;
(ii) there exists $Z\in \Omega$ independent of *Y* such that random variables *X* and $Y|(Y=Z)$ are identically distributed.

### Proof

(i) Suppose $X\prec Y$. Then by Definition 2.1 there exist a constant number $c\in {\mathbb {R}}_+$ and $Z\in \Omega$ such that $\sum \nolimits _{n=0}^\infty \frac{p_X(n)}{p_Y(n)}=c\sum \nolimits _{n=0}^\infty p_{Z}(n)=c<\infty$. Now assume that $\sum \nolimits _{n=0}^\infty \frac{p_X(n)}{p_Y(n)}:=c<\infty$. Then let *Z* be a random variable with probability mass function $p_Z(n)=\frac{p_X(n)}{cp_Y(n)},$ $n\in {\mathbb {N}}_0$. Then, obviously, $Z\in \Omega$ and we have that $p_X(n)=cp_Y(n)p_Z(n), n\in {\mathbb {N}}_0$. Hence, $X\prec Y$.
(ii) Assume that such $Z\in \Omega$ exists. Then for $n\in {\mathbb {N}}_0$
  $$\begin{aligned}&p_X(n)=P(X=n)=P(Y=n|Y=Z)=\frac{p_Y(n)p_Z(n)}{P(Y=Z)}=cp_Y(n)p_Z(n), \end{aligned}$$
  where $c=\frac{1}{P(Y=Z)}$. Hence, $X\prec Y$. Conversely, if $X\prec Y,$ then there exists $Z\in \Omega$ satisfying (15). Then taking *Z* to be independet of *Y* we get that
  $$\begin{aligned} 1=\sum _{n=0}^\infty p_X(n)=c\sum _{n=0}^\infty p_Y(n)p_Z(n)=cP(Y=Z), \end{aligned}$$
  which means that $c=\frac{1}{P(Y=Z)}$. Hence, for $n\in {\mathbb {N}}_0$
  $$\begin{aligned}&P(X=n)=p_X(n)=cp_Y(n)p_Z(n)=\frac{p_Y(n)p_Z(n)}{P(Y=Z)}=P(Y=n|Y=Z) \end{aligned}$$
  which means that *X* and $Y|(Y=Z)$ are identically distributed. $\square$

### Proposition 3.2

$\{\Omega , \preceq \}$ is a partially ordered set.

### Proof

Obviously $X\preceq X$ for any $X\in \Omega$. Now suppose that $X\preceq Y$ and $Y\preceq X,$ for some $X,Y\in \Omega$. Then if $X\ne Y$ we should have that $X\prec Y$ and $Y\prec X$ which implies that $\sum \nolimits _{n=0}^\infty \frac{p_X(n)}{p_Y(n)}<\infty$ and $\sum \nolimits _{n=0}^\infty \frac{p_Y(n)}{p_X(n)}<\infty$ which is not possible. Thus, $X=Y$. Finally, assume that $X\preceq Y$ and $Y\preceq Z,$ for some $X,Y,Z\in \Omega$. Obviously, if either $X=Y$ or $Y=Z$ then $X\preceq Z$. So let us assume that $X\prec Y$ and $Y\prec Z.$ Then $\sum \nolimits _{n=0}^\infty \frac{p_X(n)}{p_Y(n)}<\infty$ and $\sum \nolimits _{n=0}^\infty \frac{p_Y(n)}{p_Z(n)}<\infty$. Hence, $\sum \nolimits _{n=0}^\infty \frac{p_X(n)}{p_Z(n)}=\sum \nolimits _{n=0}^\infty \frac{p_X(n)}{p_Y(n)}\frac{p_Y(n)}{p_Z(n)}<\infty$ which means that $X\prec Z$ and the transitivity is shown. $\square$

Let us now check that $\prec$ does not coincide with the restriction to $\Omega$ of any well known stochastic order presented in Shaked and Shanthikumar (2007). First, as it is shown in Shaked and Shanthikumar (2007) (formulas 1.A.7, 5.A.5, theorems 1.B.1, 1.B.42, 1.C.1, 3.B.13, 5.C.1 and pp. 71, 255), for the *usual stochastic order*, *hazard rate order*, *reversed hazard order*, *likelihood ratio order*, *convolution order*, *dispersive order*, *Laplace transform order*, *factorial moments order* and the *moments order,* the fact that *X* is smaller than *Y* with respect to any of these orders implies that ${\mathbb {E}}[X]\le {\mathbb {E}}[Y]$. As for $X\sim Poisson(2)$ and $Y\sim Negative Binomial(1,1/2)$ we have that $X\prec Y$ and ${\mathbb {E}}[X]=2>1={\mathbb {E}}[Y],$ then $\preceq$ is not correlated with any of the above orders. Also, as it is shown in Shaked and Shanthikumar (2007) (formulas 2.B.7, 3.A.2, 3.A.4, 3.C.8 and p. 166) for the *mean residual life order*, *harmonic mean residual life order*, *convex order* and *excess wealth order*, the fact that *X* is smaller than *Y* with respect to any of these orders together with the condition ${\mathbb {E}}[X]={\mathbb {E}}[Y]$ implies that $var[X]\le var[Y]$. As for the random variables $X, Y\in \Omega$ with probablity mass functions defined as

$$\begin{aligned} p_{X}(n) = {\left\{ \begin{array}{ll} 1-\sum _{n=1}^\infty p_{X}(n), &{} n=0 \\ 3\sum _{n=3}^\infty \frac{1}{n^3}+2\sum _{n=3}^\infty \frac{1}{n^4}, &{} n=1 \\ \sum _{n=3}^\infty \frac{1}{n^2}+\sum _{n=3}^\infty \frac{1}{n^5}, &{} n=2\\ \frac{1}{n^6}, &{} n>2 \end{array}\right. } \end{aligned}$$

and

$$\begin{aligned} p_{Y}(n) = {\left\{ \begin{array}{ll} 1-\sum _{n=1}^\infty p_{Y}(n), &{} n=0 \\ 3\sum _{n=3}^\infty \frac{1}{n^5}+2\sum _{n=3}^\infty \frac{1}{n^2}, &{} n=1 \\ \sum _{n=3}^\infty \frac{1}{n^3}+\sum _{n=3}^\infty \frac{1}{n^4}, &{} n=2\\ \frac{1}{n^4} &{} n>2. \end{array}\right. } \end{aligned}$$

we have that $X\prec Y,$ ${\mathbb {E}}[X]={\mathbb {E}}[Y]$ but $var[X]>var[Y],$ then $\preceq$ is not correlated with any of those orders as well. Finally, as for $X\sim Poisson(1)$ and $Y\sim Negative Binomial(1,1/3)$ we have that $X\prec Y$ but ${\mathbb {E}}[e^{sX}]>{\mathbb {E}}[e^{sY}]$ at $s=\ln 2,$ then $\preceq$ is also uncorrelated with the so called *moment generating function order* (recall that given two nonnegative random variables *X* and *Y* with ${\mathbb {E}}[e^{s_0Y}]<\infty$ for some $s_0>0,$ we say that *X* is smaller than *Y* in the moment generating function order if ${\mathbb {E}}[e^{sX}]\le {\mathbb {E}}[e^{sY}]$ for all $s>0$). Thus, unlike the discrete stochastic orders presented above, $\preceq$ does not guarantee any order-preserving property for the operations of taking expected values, variances or moment generating functions. The reason for this is that $\prec$ compares whether the tails of the random variables are sufficiently far from each other and is therefore not sensitive to the probabilities of smaller values. We, therefore, offer the following proposition:

### Proposition 3.3

Let *X* and *Y* be two elements of $\Omega$ with $X\prec Y.$ Then

(i) there exists $k\in {\mathbb {N}}$ such that ${\mathbb {E}}[X^m]\le {\mathbb {E}}[Y^m]$ for all $m>k$;
(ii) there exists $s_0>0$ such that ${\mathbb {E}}[e^{sX}]\le {\mathbb {E}}[e^{sY}]$ for all $s>s_0$.

Let us recall some characteristics of partially ordered sets. Given a partially ordered set $\{P,\le \}$ an element $x\in P$ is called *minimal* if there is no element $y\in P$ such that $y\le x, y\ne x$. Similarly, an element $x\in P$ is called *maximal* if there is no element $y\in P$ such that $x\le y,x\ne y$. An element $x\in P$ is said to be *covered* by an element $y\in P$ if $x\le y,x\ne y,$ and for any $z\in P$ with $x\le z \le y$ we have that either $z=x$ or $z=y$. The next proposition deals with the above concepts for the set $\Omega$.

### Proposition 3.4

(i) There is no minimal element in $\Omega$;
(ii) an element $X\in \Omega$ is maximal if and only if $\sum \nolimits _{n=0}^\infty \sqrt{p_X(n)}=\infty$;
(iii) an element $X\in \Omega$ is covered by $Y\in \Omega$ if and only if $\sum \nolimits _{n=0}^\infty \frac{p_X(n)}{p_Y(n)}<\infty$ and $\sum \nolimits _{n=0}^\infty \sqrt{\frac{p_X(n)}{p_Y(n)}}=\infty$;
(iv) every non-maximal element $X\in \Omega$ is covered by some element $Y\in \Omega$. If additionally $\sum \nolimits _{n=0}^\infty \big (p_X(n)\big )^{1/4}=\infty ,$ then *X* is covered by some maximal element $Y\in \Omega$;
(v) for every non-maximal element $X\in \Omega$ there is a maximal element $Y\in \Omega$ such that $X\prec Y$.

The following proposition presents the closure property of $\prec$ under the operations of summation and multiplication of random variables from $\Omega$.

### Proposition 3.5

If $X_1,\ldots ,X_k$ and $Y_1,\ldots ,Y_k$ are two sets of independent random variables from $\Omega$ with $X_i\prec Y_i, i=1,\ldots ,k,$ then

(i) $$X_1+\cdots +X_k\prec Y_1+\cdots +Y_k;$$
(ii) $$X_1\cdot \cdots \cdot X_k\prec Y_1\cdot \cdots \cdot Y_k.$$

### Proof

(i) It suffices to prove that $X_1+X_2\prec Y_1+Y_2$. As $X_1\prec Y_1$ and $X_2\prec Y_2$ then $\sum \nolimits _{n=0}^\infty \frac{p_{X_1}(n)}{p_{Y_1}(n)}<\infty$ and $\sum \nolimits _{n=0}^\infty \frac{p_{X_2}(n)}{p_{Y_2}(n)}<\infty$. Therefore,
  $$\begin{aligned}&\sum \limits _{n=0}^\infty \frac{p_{X_1+X_2}(n)}{p_{Y_1+Y_2}(n)}=\sum \limits _{n=0}^\infty \frac{\sum \nolimits _{i=0}^n p_{X_1}(i)p_{X_2}(n-i)}{\sum \nolimits _{j=0}^n p_{Y_1}(j)p_{Y_2}(n-j)}\\&\quad =\sum \limits _{n=0}^\infty \sum \limits _{i=0}^n \frac{ p_{X_1}(i)p_{X_2}(n-i)}{\sum \nolimits _{j=0}^n p_{Y_1}(j)p_{Y_2}(n-j)}\\&\quad \le \sum \limits _{n=0}^\infty \sum \limits _{i=0}^n \frac{ p_{X_1}(i)p_{X_2}(n-i)}{ p_{Y_1}(i)p_{Y_2}(n-i)}=\sum \limits _{n=0}^\infty \sum \limits _{\begin{array}{c} 0\le i,j\le n \\ i+j=n \end{array}} \frac{ p_{X_1}(i)p_{X_2}(j)}{ p_{Y_1}(i)p_{Y_2}(j)}\\&\quad =\sum \limits _{i=0}^\infty \frac{p_{X_1}(i)}{p_{Y_1}(i)}\sum \limits _{j=0}^\infty \frac{p_{X_2}(j)}{p_{Y_2}(j)}<\infty , \end{aligned}$$
  where the first inequality above holds as $\sum \nolimits _{j=0}^n p_{Y_1}(j)p_{Y_2}(n-j)\ge p_{Y_1}(i)p_{Y_2}(n-i)$ for each $i=0,1,\ldots n$, and the last equality holds because for any $i,j\in {\mathbb {N}}_0,$ the summand $\frac{ p_{X_1}(i)p_{X_2}(j)}{ p_{Y_1}(i)p_{Y_2}(j)}$ appears exactly once on each side of that equality. Thus, $X_1+X_2\prec Y_1+Y_2$.
(ii) Follows from
  $$\begin{aligned}&\sum \limits _{n=0}^\infty \frac{p_{X_1\cdot X_2}(n)}{p_{Y_1\cdot Y_2}(n)}=\sum \limits _{n=0}^\infty \frac{\sum \nolimits _{k\cdot m=n} p_{X_1}(k)p_{X_2}(m)}{\sum \nolimits _{r\cdot t=n} p_{Y_1}(r)p_{Y_2}(t)}=\sum \limits _{n=0}^\infty \sum \limits _{k\cdot m=n}\frac{ p_{X_1}(k)p_{X_2}(m)}{\sum \nolimits _{r\cdot t=n} p_{Y_1}(r)p_{Y_2}(t)}\\&\quad \le \sum \limits _{n=0}^\infty \sum \limits _{k\cdot m=n} \frac{p_{X_1}(k)p_{X_2}(m)}{ p_{Y_1}(k)p_{Y_2}(m)}=\sum \limits _{k=0}^\infty \frac{p_{X_1}(k)}{p_{Y_1}(k)}\sum \limits _{m=0}^\infty \frac{p_{X_2}(m)}{p_{Y_2}(m)}<\infty . \end{aligned}$$

$$\square $$

Note that if $X\in \Omega$ then $p_{X^2}(2)=0$ and if $a\ne 1$ is some real number, then the support of *aX* is the set $a{\mathbb {N}}_0\ne {\mathbb {N}}_0$. Hence, as the support of all elements of $\Omega$ is ${\mathbb {N}}_0,$ we get that neither $X^2$ nor *aX* belong to $\Omega$. However, $\prec$ is closed under the permutations of the support set of elements of $\Omega$. More precisely, let $\sigma$ be any permutation of ${\mathbb {N}}_0$, that is, a one-to-one mapping from ${\mathbb {N}}_0$ to ${\mathbb {N}}_0$. Then for $X\in \Omega ,$ let $X_\sigma$ be an element of $\Omega$ with probability mass function $p_{X_\sigma }(n)=p_X(\sigma (n)), n\in {\mathbb {N}}_0$.

### Proposition 3.6

Let $\sigma$ be any permutation of ${\mathbb {N}}_0.$ Then $X\prec Y$ if and only if $X_\sigma \prec Y_\sigma .$

### Proof

As convergent series with positive terms converge unconditionally, then $\sum \nolimits _{n=0}^\infty p_X(n)/p_Y(n)<\infty$ if and only if $\sum \nolimits _{n=0}^\infty p_X(\sigma (n))/p_Y(\sigma (n))<\infty .$ $\square$

The survival function of a random variable $X\in \Omega$ is given by

$$\begin{aligned} R_X(n)=\sum \limits _{ k> n}p_X(k), \quad n\in {\mathbb {N}}_0. \end{aligned}$$

As $X\prec Y$ indicates that the tail of *Y* is heavier than the tail of *X*,  and the survival function gives the probability of the right tail $(n,\infty ),$ then $X\prec Y$ should imply that $R_X(n)$ is smaller than $R_Y(n)$. In fact we have the following:

### Theorem 3.1

If $X,Y\in \Omega$ and $X\prec Y$ then

16 $$\begin{aligned} \lim \limits _{n\rightarrow +\infty }\frac{R_X(n)}{R_Y(n)}=0. \end{aligned}$$

### Proof

As $X,Y\in \Omega$ then $p_X(k)>0$ and $p_Y(k)>0$ for all $k\in {\mathbb {N}}_0$. This means that $\{R_X(n)\}_{n\in {\mathbb {N}}}$ and $\{R_Y(n)\}_{n\in {\mathbb {N}}}$ are both strictly decreasing sequences converging to 0. As $X\prec Y$ then $\sum \nolimits _{n=0}^\infty p_X(n)/p_Y(n)<\infty ,$ and, therefore,

$$\begin{aligned} \lim \limits _{n\rightarrow \infty }\frac{R_X(n+1)-R_X(n)}{R_Y(n+1)-R_Y(n)}=\lim \limits _{n\rightarrow \infty }\frac{p_X(n+1)}{p_Y(n+1)}=0. \end{aligned}$$

Hence, by the Stolz–Cesàro theorem (see, e.g., Choudary and Niculescu (2014), Theorem 2.7.1) we get (16). $\square$

Note, however, that the converse is not necessarily true, that is, (16) does not necessarily imply $X\prec Y$. In fact, as the following example shows, even the condition $\sum \nolimits _{n=0}^\infty \frac{R_X(n)}{R_Y(n)}<\infty ,$ which is stronger than (16), does not necessarily imply that $\frac{p_X(n)}{p_Y(n)}$ is bounded from above (which is much weaker than $X\prec Y$).

### Example 3.1

Consider $$X,Y\in \Omega $$ with

$$\begin{aligned} p_{X}(k)= & {} {\left\{ \begin{array}{ll} 1-\sum \limits _{k=1}^\infty p_{X}(k), &{} k=0 \\ 5^{-k-1}, &{} k>0 \end{array}\right. }\\ p_{Y}(k)= & {} {\left\{ \begin{array}{ll} 1-\sum \limits _{k=1}^\infty p_{Y}(k), &{} k=0 \\ 2^{-k-1}, &{} \;k\; \text {is odd} \\ 6^{-k-1}, &{} k>0, \;k\; \text {is even.} \\ \end{array}\right. } \end{aligned}$$

Then, as

$$\begin{aligned} R_X(n)=\sum \limits _{ k> n}p_X(k)=\sum \limits _{ k> n}5^{-k-1}=\frac{5^{-n}}{20} \end{aligned}$$

and

$$\begin{aligned} R_Y(n)=\sum \limits _{ k> n}p_X(k)>\sum \limits _{\begin{array}{c} k>n \\ k\; \text {is odd} \end{array}}p_X(k)=\sum \limits _{\begin{array}{c} k>n \\ k\; \text {is odd} \end{array}}2^{-k-1}> \sum \limits _{ k> n}4^{-k-1}=\frac{4^{-n}}{12}, \end{aligned}$$

we have that $\sum \nolimits _{n=0}^\infty \frac{R_X(n)}{R_Y(n)}<\infty .$ However, $\frac{p_X(2n)}{p_Y(2n)}=(6/5)^{2n+1}\rightarrow \infty$ as $n\rightarrow \infty$.

Note also that in general (16) is the best that we can achieve in Theorem 3.1. More precisely, as the following example shows, the condition $X\prec Y$ alone does not necessarily imply

17 $$\begin{aligned} \sum \limits _{n=0}^\infty \frac{R_X(n)}{R_Y(n)}<\infty . \end{aligned}$$.

### Example 3.2

Consider $$X,Y\in \Omega $$ with

$$\begin{aligned} p_{X}(k)= & {} {\left\{ \begin{array}{ll} 1-\sum \limits _{k=1}^\infty p_{X}(k), &{} k=0 \\ \frac{1}{10}\frac{1}{n^2n!}, &{} k=n^2, n>0\\ \frac{1}{10}\frac{1}{4^k(n+1)!}, &{} n^2<k<(n+1)^2, n>0 \end{array}\right. }\\ p_{Y}(k)= & {} {\left\{ \begin{array}{ll} 1-\sum \limits _{k=1}^\infty p_{Y}(k), &{} k=0 \\ \frac{1}{10}\frac{1}{n!}, &{} k=n^2, n>0\\ \frac{1}{10}\frac{1}{2^k(n+1)!}, &{} n^2<k<(n+1)^2, n>0. \end{array}\right. } \end{aligned}$$

Then, clearly, $\sum \nolimits _{k=0}^\infty p_X(k)/p_Y(k)<\infty$ and $X \prec Y$. For $n>1$ we have that

$$\begin{aligned} R_Y(n^2)&=\sum \limits _{k>n^2}p_Y(k)=\sum \limits _{m=n}^\infty \sum \limits _{k=m^2+1}^{(m+1)^2}p_Y(k)\le \sum \limits _{m=n}^\infty \frac{1}{(m+1)!}\\&\quad<\frac{1}{(n+1)!}\sum \limits _{m=0}^\infty \frac{1}{n^m}<\frac{3}{(n+1)!}. \end{aligned}$$

Hence,

$$\begin{aligned}&\sum \limits _{n=0}^\infty \frac{R_X(n)}{R_Y(n)}=\sum \limits _{k=0}^\infty \sum \limits _{n=k^2}^{(k+1)^2-1} \frac{R_X(n)}{R_Y(n)}>\sum \limits _{k=2}^\infty \sum \limits _{n=k^2}^{(k+1)^2-1} \frac{p_X((k+1)^2)}{R_Y(n)}\\&\quad>\sum \limits _{k=2}^\infty \big [(k+1)^2-k^2\big ]\frac{p_X((k+1)^2)}{R_Y(k^2)}>\frac{1}{30}\sum \limits _{k=2}^\infty \frac{2k+1}{(k+1)^2} \end{aligned}$$

and the last series diverges.

However, (17) holds under the additional assumption of the existence of a positive constant $c>0$ such that

18 $$\begin{aligned} p_X(k)/p_Y(k)\le cp_X(n)/p_Y(n) \end{aligned}$$

for all $0\le n<k$. In particular, if the sequence $\{p_X(n)/p_Y(n)\}_{n=0}^\infty$ is eventually non-increasing then (18) is satisfied.

### Proposition 3.7

If $X,Y\in \Omega$ satisfy (18) and $X\prec Y$ then

$$\begin{aligned} \sum \limits _{n=0}^\infty \frac{R_X(n)}{R_Y(n)}<\infty . \end{aligned}$$

### Proof

Follows from

19 $$\begin{aligned}&\sum \limits _{n=0}^\infty \frac{R_X(n)}{R_Y(n)}=\sum \limits _{n=0}^\infty \frac{\sum \limits _{ k> n}p_X(k)}{\sum \limits _{ k> n}p_Y(k)}=\sum \limits _{n=0}^\infty \frac{\sum \limits _{ k> n}p_Y(k)p_X(k)/p_Y(k)}{\sum \limits _{ k> n}p_Y(k)}\nonumber \\&\quad \le \sum \limits _{n=0}^\infty \frac{cp_X(n)/p_Y(n)\sum \limits _{ k> n}p_Y(k)}{\sum \limits _{ k> n}p_Y(k)}=c\sum \limits _{n=0}^\infty \frac{p_X(n)}{p_Y(n)}<\infty . \end{aligned}$$

$$\square $$

## Compound distributions

Consider the compound distribution

$$\begin{aligned} S=M_1+\cdots +M_N, \end{aligned}$$

where the counting random variable *N* represents the number of claims and $M_1,M_2,\ldots$ represent the claim size. It is assumed that $M_1,M_2,\ldots$ are independent and identically distributed discrete random variables which do not depend on *N* and that $S=0$ if $N=0$. Denoting $p(n)=P(N=n)$, $g_n=P(S=n)$ and $h_n=P(M=n)$ we have that

$$\begin{aligned} g_k=\sum \limits _{n=1}^\infty p(n)h_k^{*n}, \end{aligned}$$

where $h_k^{*n}=P(M_1+\cdots +M_n=k)$ is the *n*-fold convolution of $h_k$, $k\in {\mathbb {N}},$ and $g_0=\sum \nolimits _{n=0}^\infty p(n)h_0^{*n}=\sum \nolimits _{n=0}^\infty p(n)h_0^{n}=G_N(h_0)$. The probability generating function of *S* is given by

$$\begin{aligned} G_S(z)=G_N(G_M(z)), \end{aligned}$$

where $G_M(z)$ is the common probability generating function of $M_1,M_2,\ldots$ and $G_N(z)$ is the probability generating function of *N*.

As we want to compare compound distributions with respect to $\prec ,$ then, in order to guarantee that the resulting compound distributions are from $\Omega ,$ i.e., that their support set is ${\mathbb {N}}_0$, we will assume that $M_1,M_2\ldots$ are all from $\Omega$. In order to compare distributions with respect to $\prec$ we need to know the asymptotic behaviour of their probability mass functions. In general, estimating the asymptotic behaviour of compound distributions is not easy. However, under certain assumptions on claim number and claim size distributions various asymptotic estimates for compound distributions have been obtained (see, e.g., Embrechts et al. (1984) and its references). We first apply the following theorem from Chover et al. (1973).

### Theorem 4.1

Let *M* be the common distribution of $M_1,M_2,\ldots$ and assume that the following conditions hold:

(i) $$\lim \limits _{n\rightarrow \infty }\frac{h_n^{*2}}{h_n}=c<\infty $$;
(ii) $$\lim \limits _{n\rightarrow \infty }\frac{h_{n+1}}{h_n}=\frac{1}{r}>0$$;
(iii) $G_M(r)=d<\infty$ and $G_N(z)$ is analytic in a region containing the range of $G_M(z)$ for $|z|\le r$.

Then

$$\begin{aligned} \lim \limits _{n\rightarrow +\infty }\frac{g_n}{h_n}=G^\prime _N(d). \end{aligned}$$

As described in Chover et al. (1973), the above conditions are satisfied by many examples. We then have the following proposition.

### Proposition 4.1

Let $S'=M'_1+\cdots +M'_{N'}$ and $S''=M''_1+\cdots +M''_{N''},$ where $M'_1,M'_2,\ldots$ as well as $M''_1,M''_2,\ldots$ are independent and identically distributed random variables from $\Omega$ following common distributions $M'$ and $M''$ respectively. Assume that the pairs $\{M',N'\}$ and $\{M'',N''\}$ satisfy the conditions of above theorem. Then $S'\prec S''$ if and only if $M'\prec M''.$

Let us now consider mixed Poisson distributions. In this case

$$\begin{aligned} g_n=\int \limits _0^\infty \frac{(\lambda x)^ne^{-\lambda x}}{n!}f(x)d\mu (x), \quad n\in {\mathbb {N}}_0, \end{aligned}$$

where $\mu$ is Lebesgue or counting measure with generalized density *f*(*x*). It is shown in Willmot (1990) that if $f(x)\sim C(x)x^\alpha e^{-\beta x},$ as $x\rightarrow \infty$, where *C*(*x*) is a locally bounded function on $(0, \infty )$ which varies slowly at infinity, $\beta \ge 0$ and $-\infty<\alpha <\infty ,$ then

$$\begin{aligned} g_n\sim \frac{C(n)}{(\lambda +\beta )^{\alpha +1}}\Big (\frac{\lambda }{\lambda +\beta }\Big )^nn^\alpha . \end{aligned}$$

Clearly $Poisson(\lambda _1)\prec Poisson(\lambda _2)$ if and only if $\lambda _1<\lambda _2$ in which case $\frac{\lambda _1}{\lambda _1+\beta }<\frac{\lambda _2}{\lambda _2+\beta }$ for all $\beta >0$. Thus, if the densities $f_1$ and $f_2$ in both mixtures share the same $\beta >0,$ then $Poisson(\lambda _1)\prec Poisson(\lambda _2)$ implies $S_1\prec S_2,$ where $S_1\sim (\lambda _1,f_1)$ and $S_2\sim (\lambda _2,f_2)$. However, if $\beta =0$ (in which case we should have $\alpha <-1$), then the condition $Poisson(\lambda _1)\prec Poisson(\lambda _2)$ alone does not imply $S_1\prec S_2.$ In order to have $S_1\prec S_2$ in this case, we should instead assume that $f_1$ and $f_2$ are such that $\sum \nolimits _{n=0}^\infty f_1(n)/f_2(n)<\infty$ which, in case of $f_1,f_2\in \Omega$, means $f_1\prec f_2$.

Recall from the introductory section, that the recursive formulae and other properties of compound distributions are usually obtained under the assumption that the corresponding claim number distribution belongs to Panjer’s family or to its certain extensions. As the order $\prec$ is obtained by an extension of Panjer’s family of distributions, then, in some sense, $\prec$ is more naturally connected to the claim number distribution *N* rather than to the claim size distribution *M*. It is therefore interesting to see when the condition $N'\prec N''$ alone implies $S'\prec S''$ given that the claim size distributions are same: $M'=M'':=M$. The following proposition shows that the desired implication holds for a large class of claim number distributions.

### Proposition 4.2

Suppose

$$\begin{aligned} p_{N'}(n)\sim C_1n^{\alpha _1}\theta _1^n \end{aligned}$$

and

$$\begin{aligned} p_{N''}(n)\sim C_2n^{\alpha _2}\theta _2^n, \end{aligned}$$

where $C_1$ and $C_2$ are some positive constants, $\alpha _1,\alpha _2\in {\mathbb {R}}$ and $\theta _1,\theta _2\in (0,1).$ Assume also that the probability generating function $G_M(z)$ of *M* has radius of convergence exceeding both $\tau _1$ and $\tau _2$, where $G_M(\tau _1)=\theta _1^{-1}$ and $G_M(\tau _2)=\theta _2^{-1}.$ Then $S'\prec S''$ if and only if $N'\prec N''.$

### Proof

As it is shown in Willmot (1989), in this case we have that

$$\begin{aligned} g'_n\sim \frac{C_1 n^{\alpha _1}\tau _1^{-n}}{\big (\theta _1\tau _1 G_M^\prime (\tau _1)\big )^{\alpha _1+1}} \end{aligned}$$

and

$$\begin{aligned} g''_n\sim \frac{C_2 n^{\alpha _2}\tau _2^{-n}}{\big (\theta _2\tau _2 G_M^\prime (\tau _2)\big )^{\alpha _2+1}}, \end{aligned}$$

where $g'_n=P(S'=n)$ and $g''_n=P(S''=n)$. Note that $N'\prec N''$ if and only if either $\theta _1<\theta _2$ or $\theta _1=\theta _2$ and $\alpha _2-\alpha _1>1$. As $G_M$ is increasing on ${\mathbb {R}}_+$, then in the first case we have that $\tau _1>\tau _2$ and, therefore, $S'\prec S''$. Clearly in the second case we again have that $S'\prec S''$. $\square$

The above argument can also be applied to get the desired implication in the cases when one of the claim number distributions is asymptotically smaller (e.g. Poisson distribution) or larger (e.g. Waring distribution) than $n^\alpha \theta ^n$.

To conclude this section, we note that in general the conditions $M'\prec M''$ and $N'\prec N''$ do not necessarily imply $S'\prec S''$. To see this let $p_{N'}(n)\sim C_14^{-n}, p_{N''}(n)\sim C_23^{-n}$, $M'\sim Poisson(\ln 4)$ and $M''\sim NB(1,1/4)$. Then $G_{M'}(2)=4$ and $G_{M''}(3)=3$ and, therefore, $g'_n\sim C_1 2^{-n}$ and $g''_n\sim C_2 3^{-n}$. Hence, not only does $S'\prec S''$ not hold but in fact $S''\prec S'$.

## Generation of new families of distributions

In this section we will consider the following distributions from $\Omega :$

**Table:** 

Distribution	*p*(*n*), $n\in {\mathbb {N}}_0$	Parameters
Poisson	$\frac{e^{-\lambda }\lambda ^n}{n!}$	$\lambda >0$
Hyper-Poisson	$\frac{\Gamma (\eta )\mu ^n}{\Gamma (\eta +n)M(1,\eta ,\mu )}$	$\mu >0$, $\eta >0$
Negative Binomial	$\frac{\Gamma (n+r)(1-p)^rp^n}{n!\Gamma (r)}$	$r>0$, $0<p<1$
Shifted Logarithmic	$\frac{\theta ^{n+1}}{-(n+1)\log (1-\theta )}$	$0<\theta <1$
Waring	$\frac{\alpha \Gamma (p+n)\Gamma (\alpha +p)}{\Gamma (p)\Gamma (\alpha +p+n+1)}$	$\alpha >0$, $p>0$
Poisson-Beta	$\frac{\nu ^n}{n!}\frac{\Gamma (\alpha +\beta )}{\Gamma (\alpha )}\sum \limits _{j=0}^\infty \frac{\Gamma (\alpha +n+j)}{\Gamma (\alpha +\beta +n+j)}\frac{(-\nu )^j}{j!}$	$\nu>0, \alpha>0, \beta >0$

We will use the notations^1^$X_p, X_{hp}, X_{nb}, X_{sl}$, $X_w$ and $X_{pb}$ to denote random variables following Poisson, Hyper-Poisson, Negative Binomial, Shifted Logarithmic, Waring and Poisson-Beta distributions respectively.

As was mentioned in the first section, the probability mass functions of the Poisson and Negative Binomial distributions satisfy the recurrence relation

20 $$\begin{aligned} p(n)=\frac{a_0n+b_0}{n}p(n-1), \quad n\in {\mathbb {N}}. \end{aligned}$$

It is also known (see Willmot and Panjer 1987), that the probability mass functions of the Hyper-Poisson, Shifted Logarithmic and Waring distributions satisfy the first order linear recursion

21 $$\begin{aligned} p(n)=\frac{a_1n+b_1}{c_1n+1}p(n-1), \quad n\in {\mathbb {N}}, \end{aligned}$$

and the probability mass function of the Poisson-Beta distribution, together with some other distributions, such as Poisson-Generalized Inverse Gaussian distribution, satisfies the second order quadratic recursion

22 $$\begin{aligned} p(n)=\frac{a_2n+b_2}{n}p(n-1)+\frac{c_2n+d_2}{n(n-1)}p(n-2), \quad n>1, \end{aligned}$$

where the values of the coefficients are determined by the values of the parameters of the corresponding distributions. As for any $\alpha >0$

$$\begin{aligned} \lim \limits _{n\rightarrow \infty }\frac{\Gamma (n+\alpha )}{\Gamma (n)n^\alpha }=1, \end{aligned}$$

we have the following:

### Proposition 5.1

(i) if $$\nu <\lambda $$ then $$X_{pb}(\nu ,\alpha ,\beta )\prec X_{p}(\lambda )$$;
(ii) if $\nu <\mu$ then $X_{pb}(\nu ,\alpha ,\beta )\prec X_{hp}(\mu , \eta )$;
(iii) for any values of parameters of corresponding distributions, $X_{pb}$ is $\prec$ than each of $X_{nb},$ $X_{sl}$ and $X_{w}$.

Now let *X* be an element of $\Omega$ with probability mass function satisfying the first order recursion

23 $$\begin{aligned} p_X(n)=f(n)p_X(n-1), \quad n\in {\mathbb {N}}, \end{aligned}$$

and let *Y* be an element of $\Omega$ with probability mass function satisfying the second order recursion

24 $$\begin{aligned} p_Y(n)=g(n)p_Y(n-1)+h(n)p_Y(n-2), \quad n>1, \end{aligned}$$

where *f*, *g* and *h* are some functions from ${\mathbb {N}}$ to ${\mathbb {R}}.$ Then, if $Y\prec X,$ then for the random variable *Z* with

$$\begin{aligned} p_{Y}(n)=cp_{X}(n)p_Z(n), \quad n\in {\mathbb {N}}_0, \end{aligned}$$

we have that the probability mass function of *Z* satisfies the following second order recursion:

25 $$\begin{aligned} \begin{aligned}&p_Z(n)=\frac{p_{Y}(n)}{cp_X(n)}=\frac{g(n)}{f(n)}p_Z(n-1)+\frac{h(n)}{f(n)f(n-1)}p_Z(n-2), \quad n>1. \end{aligned} \end{aligned}$$

Rewriting the probability mass function of the Poisson-Beta distribution in terms of the confluent hypergeometric function *M* as

$$\begin{aligned} p_{pb}(n)=\frac{\nu ^n}{n!}\frac{\Gamma (\alpha +\beta )}{\Gamma (\alpha )}\frac{\Gamma (\alpha +n)}{\Gamma (\alpha +\beta +n)}M(\alpha +n,\alpha +\beta +n,-\nu ) \end{aligned}$$

and using asymptotic properties of *M* (see, e.g., NIST Digital Library of Mathematical Functions, Ch. 13) we get that

$$\begin{aligned} p_{pb}(n)=\frac{\nu ^n}{n!}\frac{e^{-\nu }}{n^\beta }\frac{\Gamma (\alpha +\beta )}{\Gamma (\alpha )}\bigg (1+O\bigg (\frac{1}{n}\bigg )\bigg ). \end{aligned}$$

We therefore have the following:

### Proposition 5.2

(i) if $$\lambda <\nu $$ then $$X_{p}(\lambda )\prec X_{pb}(\nu ,\alpha ,\beta )$$;
(ii) if $\mu <\nu$ then $X_{hp}(\mu , \eta )\prec X_{pb}(\nu ,\alpha ,\beta )$.

Now assume that *X* is an element of $\Omega$ with probability mass function satisfying (23), *Y* is an element of $\Omega$ with probability mass function satisfying (24), and $X\prec Y$. Then for the random variable *Z* with

$$\begin{aligned} p_{X}(n)=cp_{Y}(n)p_Z(n), \quad n\in {\mathbb {N}}_0, \end{aligned}$$

we have that the probability mass function of *Z* satisfies the following second order inverse recursion:

26 $$\begin{aligned} \begin{aligned}&\frac{1}{p_Z(n)}=\frac{cp_{Y}(n)}{p_X(n)}=\frac{g(n)}{f(n)}\frac{1}{p_Z(n-1)}+\frac{h(n)}{f(n)f(n-1)}\frac{1}{p_Z(n-2)}, \quad n>1. \end{aligned} \end{aligned}$$

### Recursion formula

Let *X* belong to the family of distributions satisfying (20) and let *Y* belong to the family of distributions satisfying (22). Then *X* follows either Poisson or Negative Binomial distribution. In the first case we have that in (20) $a_0=0$ and applying (25) we get that the probability mass function of *Z* satisfies the reccurence relation

27 $$\begin{aligned} p(n)=\frac{1}{b_0}(a_2n+b_2)p(n-1)+\frac{1}{b_0^2}(c_2n+d_2)p(n-2), \quad n>1. \end{aligned}$$

Dividing both sides of the above expression by $n+1$ we can rewrite (27) as

$$\begin{aligned}&\frac{1}{n+1}p(n)=\bigg [\frac{a_2}{b_0}+\frac{b_2-a_2}{b_0(n+1)}\bigg ]p(n-1)+\bigg [\frac{c_2}{b_0^2}+\frac{d_2-c_2}{b_0^2(n+1)}\bigg ]p(n-2), \quad n>1. \end{aligned}$$

If *X* follows a Negative Binomial distribution, then in (20) $a_0\ne 0$ and applying (25) we get

28 $$\begin{aligned} (a_0n+b_0)(a_0n-a_0+b_0)p(n)= & {} (a_2n+b_2)(a_0n-a_0+b_0)p(n-1)\nonumber \\&+\,(c_2n+d_2)p(n-2), \quad n>1. \end{aligned}$$

As the coefficients of each of $p(n), p(n-1)$ and $p(n-2)$ in the above expression are polynomials on *n* of degree at most 2, then each of them can be written in the form $d_1(n+1)(n+2)+d_2(n+1)+d_3,$ where for each case the values of the fixed terms $d_1, d_2$ and $d_3$ are determined by the values of corresponding fixed terms appearing in (28). Using this decomposition and dividing both sides of (28) by $(n+1)(n+2)$ (also using $\frac{1}{(n+1)(n+2)}=\frac{1}{n+1}-\frac{1}{n+2}$), we get that (28) can be rewritten as

$$\begin{aligned}&\bigg [a_0^2+\frac{2a_0^2+b_0^2-a_0b_0}{n+1}+\frac{3a_0b_0-6a_0^2-b_0^2}{n+2}\bigg ]p(n)\\&\quad =\bigg [a_0a_2+\frac{2a_0a_2+b_0b_2-2a_0b_2-a_2b_0}{n+1}+\frac{2a_2b_0-6a_0a_2+3a_0b_2-b_0b_2}{n+2}\bigg ]\\&p(n-1)\\&\qquad +\bigg [\frac{d_2-c_2}{n+1}+\frac{2c_2-d_2}{n+2}\bigg ]p(n-2), \quad n>1. \end{aligned}$$

Thus, in both cases we get that the probability mass function of *Z* satisfies the recurrence relation

29 $$\begin{aligned}&\bigg [A+\frac{B}{n+1}+\frac{C}{n+2}\bigg ]p(n) \nonumber \\&\quad =\bigg [D+\frac{E}{n+1}+\frac{F}{n+2}\bigg ]p(n-1)\nonumber \\&\qquad +\bigg [G+\frac{H}{n+1}+\frac{J}{n+2}\bigg ]p(n-2), \quad n>1. \end{aligned}$$

Now assume that the counting random variable *N* with $p(n)=P(N=n)$ satisfies (29) and consider the compound distribution

$$\begin{aligned} S=M_1+\cdots +M_N, \end{aligned}$$

where, as before, claim sizes $M_1,M_2,\ldots$ are independent and identically distributed discrete random variables which are independent of *N* and follow a common distribution *M*, $h_k=P(M=k)$ and

$$\begin{aligned} g_k=P(S=k)=\sum \limits _{n=1}^\infty p(n)h_k^{*n}, \end{aligned}$$

with $g_0=G_N(h_0)$. If $k>0,$ then multiplying both sides of (29) by $h_k^{*n+2}$ and summing over $n\ge 2$ yields

30 $$\begin{aligned} \begin{aligned}&A\sum \limits _{n=0}^\infty p(n+2)h_k^{*n+4}+B\sum \limits _{n=0}^\infty p(n+2)\frac{h_k^{*n+4}}{n+3}+C\sum \limits _{n=0}^\infty p(n+2)\frac{h_k^{*n+4}}{n+4}\\&\quad =D\sum \limits _{n=0}^\infty p(n+1)h_k^{*n+4}+E\sum \limits _{n=0}^\infty p(n+1)\frac{h_k^{*n+4}}{n+3}+F\sum \limits _{n=0}^\infty p(n+1)\frac{h_k^{*n+4}}{n+4}\\&\qquad +G\sum \limits _{n=0}^\infty p(n)h_k^{*n+4}+H\sum \limits _{n=0}^\infty p(n)\frac{h_k^{*n+4}}{n+3}+J\sum \limits _{n=0}^\infty p(n)\frac{h_k^{*n+4}}{n+4}. \end{aligned}\nonumber \\ \end{aligned}$$

Denoting

$$\begin{aligned} P_i(t)=\sum \limits _{n=0}^\infty p(n)\frac{t^{n+i}}{n+i}, \quad i=1,2,3, \end{aligned}$$

and applying formulae (29)–(31) from Kitano et al. (2005) together with

$$\begin{aligned} h_k^{*n+i}=\sum \limits _{j=0}^k h_j^{*i}h_{k-j}^{*n} \end{aligned}$$

and

$$\begin{aligned} \frac{h_k^{*n+i}}{n+i}=\frac{1}{ik}\sum \limits _{j=0}^k jh_j^{*i}h_{k-j}^{*n}, \end{aligned}$$

$$0<i\le 4,$$ we get

31 $$\begin{aligned}&\sum \limits _{n=0}^\infty p(n+2)h_k^{*n+4}=\sum \limits _{n=0}^\infty p(n)h_k^{*n+2}-p(0)h_k^{*2}-p(1)h_k^{*3}\nonumber \\&\quad =\sum \limits _{j=0}^k h_j^{*2}g_{k-j}-p(0)h_k^{*2}-p(1)h_k^{*3}, \end{aligned}$$

32 $$\begin{aligned}&\sum \limits _{n=0}^\infty p(n+2)\frac{h_k^{*n+4}}{n+3}=\sum \limits _{n=0}^\infty \frac{p(n)}{n+1}h_k^{*n+2}-p(0)h_k^{*2}-p(1)\frac{h_k^{*3}}{2}\nonumber \\&\quad =\sum \limits _{j=0}^{k-1}\sum \limits _{i=1}^{k-j}\frac{i}{k-j}h_jh_ig_{k-j-i}+h_kP_1(h_0)-p(0)h_k^{*2}-p(1)\frac{h_k^{*3}}{2}, \end{aligned}$$

33 $$\begin{aligned}&\sum \limits _{n=0}^\infty p(n+2)\frac{h_k^{*n+4}}{n+4}=\sum \limits _{n=0}^\infty \frac{p(n)}{n+2}h_k^{*n+2}-p(0)\frac{h_k^{*2}}{2}-p(1)\frac{h_k^{*3}}{3}\nonumber \\&\quad =\frac{1}{2k}\sum \limits _{j=0}^k jh_j^{*2}g_{k-j}-p(0)\frac{h_k^{*2}}{2}-p(1)\frac{h_k^{*3}}{3}, \end{aligned}$$

34 $$\begin{aligned}&\sum \limits _{n=0}^\infty p(n+1)h_k^{*n+4}=\sum \limits _{j=0}^k h_j^{*3}g_{k-j}-p(0)h_k^{*4}, \end{aligned}$$

35 $$\begin{aligned}&\sum \limits _{n=0}^\infty p(n+1)\frac{h_k^{*n+4}}{n+3}=\sum \limits _{j=0}^{k-1}\sum \limits _{i=1}^{k-j}\frac{i}{2(k-j)}h_jh_i^{*2}g_{k-j-i}\nonumber \\&\quad +h_kP_2(h_0)-p(0)\frac{h_k^{*3}}{2}, \end{aligned}$$

36 $$\begin{aligned}&\sum \limits _{n=0}^\infty p(n+1)\frac{h_k^{*n+4}}{n+4}=\frac{1}{3k}\sum \limits _{j=0}^k jh_j^{*3}g_{k-j}-p(0)\frac{h_k^{*3}}{3}, \end{aligned}$$

37 $$\begin{aligned}&\sum \limits _{n=0}^\infty p(n)h_k^{*n+4}=\sum \limits _{j=0}^k h_j^{*4}g_{k-j}, \end{aligned}$$

38 $$\begin{aligned}&\sum \limits _{n=0}^\infty p(n)\frac{h_k^{*n+4}}{n+3}=\sum \limits _{j=0}^{k-1}\sum \limits _{i=1}^{k-j}\frac{i}{3(k-j)}h_jh_i^{*3}g_{k-j-i}+h_kP_3(h_0), \end{aligned}$$

39 $$\begin{aligned}&\sum \limits _{n=0}^\infty p(n)\frac{h_k^{*n+4}}{n+4}=\frac{1}{4k}\sum \limits _{j=0}^k jh_j^{*4}g_{k-j}. \end{aligned}$$

Substituting (31)–(39) into (30) and solving for $g_k$ yields

40 $$\begin{aligned} \begin{aligned} g_k&=\frac{1}{Dh_0^3+Hh_0^4-Ah_0^2}\Bigg \{\sum \limits _{j=1}^k g_{k-j}\Big [Ah_j^{*2}+\frac{C}{2k}jh_j^{*2}-Dh_j^{*3}-\frac{G}{3k}jh_j^{*3}\\&\qquad -Hh_j^{*4}-\frac{K}{4k}jh_j^{*4}\Big ]\\&\quad +\sum \limits _{j=0}^{k-1}\sum \limits _{i=1}^{k-j}\frac{i}{k-j}g_{k-j-i}\Big [Bh_jh_i-Fh_ih_j^{*2}-\frac{J}{3}h_jh_i^{*3}\Big ]\\&\quad -p(0)\Big [Ah_k^{*2}+Bh_k^{*2}+C\frac{h_k^{*2}}{2}-Dh_k^{*4}-Fh_k^{*3}-G\frac{h_k^{*3}}{3}\Big ]\\&\quad -p(1)\Big [Ah_k^{*3}+B\frac{h_k^{*3}}{2}+C\frac{h_k^{*3}}{3}\Big ]+Bh_kP_1(h_0)-Fh_k^{*2}P_1(h_0)-Jh_kP_3(h_0)\Bigg \}. \end{aligned} \end{aligned}$$

In conclusion of this section we note that while the recurrence formula for the compound distributions with counting distrbution satisfying (26) cannot be obtained using the very same technique as above, it may still be interesting to derive properties of those distributions by employing different techniques.

## Applications

(i) *Applications to reliability theory.* Recall, that the *hazard function* (also called *instantaneous failure rate*) of a continuous random variable *X* with density function *f*(*x*) is defined as

$$\begin{aligned} h(t)=\frac{f(t)}{R(t)}=-\frac{d}{dt}\ln R(t), \end{aligned}$$

where $R(t)=P(X>t)$ is the survival function. The *cumulative hazard function* of *X* can then be written as

41 $$\begin{aligned} H(x):=\int _0^xh(t)dt=-\ln R(x). \end{aligned}$$

The cumulative hazard function measures the risk of failure by time *x*. For discrete random variables the hazard function is usually defined as

$$\begin{aligned} r_X(k)=\frac{p_X(k)}{R_X(k-1)}, \end{aligned}$$

and, in order to preserve the relation (41) between the cumulative hazard function and the survival function for the discrete case, some authors (see, e.g., Cox and Oakes 1984) define the discrete cumulative hazard function as

$$\begin{aligned} H_X(n):=-\sum \limits _{k\le n}\ln [1-r_X(k)]=-\ln R_X(n). \end{aligned}$$

Alternatively, the relation (41) can be preserved in the discrete case by (re)defining the hazard function of a discrete random variable *X* as

$$\begin{aligned} r^*_X(k)=-\big [\ln R_X(k)-\ln R_X(k-1)\big ]=\ln \frac{R_X(k-1)}{R_X(k)}, \end{aligned}$$

as it is done in Xie et al. (2002). The *discrete cumulative hazard function* is then given by

$$\begin{aligned} H_X(n):=\sum \limits _{k=0}^nr^*_X(k)=\ln R_X(-1)-\ln R_X(n)=-\ln R_X(n), \end{aligned}$$

as $R_X(-1)=1$. Clearly, $\lim \nolimits _{n\rightarrow \infty } H_X(n)=\infty .$ Let us now check that if $X,Y\in \Omega$ and $X\prec Y$ then $H_X(n)$ is asymptotically much bigger than $H_Y(n)$. Indeed, by Theorem 3.1,

$$\begin{aligned} H_X(n)-H_Y(n)=-\ln R_X(n)+\ln R_Y(n)=\ln \frac{R_Y(n)}{R_X(n)}\rightarrow \infty , \end{aligned}$$

as $$n\rightarrow \infty $$.

The *reverse hazard function* of $X\in \Omega$ is defined as (see Chechile 2011)

$$\begin{aligned} \lambda _{X}(n) = {\left\{ \begin{array}{ll} 1, &{} n=0 \\ \frac{p_X(n)}{F_X(n)}, &{} n>0, \end{array}\right. } \end{aligned}$$

and it gives the conditional probability of the failure at the *n*-th event given that failure occurs at $k\le n$. As $p_X(0)\le F_X(n)\le 1$ and $p_Y(0)\le F_Y(n)\le 1$, $n\in {\mathbb {N}}_0,$ we have that $X\prec Y$ if and only if $\sum \nolimits _{n=0}^\infty \frac{\lambda _X(n)}{\lambda _Y(n)}<\infty$. In particular, if $X\prec Y,$ then

$$\begin{aligned} \lim \limits _{n\rightarrow \infty }\frac{\lambda _X(n)}{\lambda _Y(n)}=0. \end{aligned}$$

For $X\in \Omega ,$ the odds for surviving age *n* is given by the *odds function for survival*

$$\begin{aligned} O_X(n)=\frac{P(X>n)}{P(X\le n)}=\frac{R_X(n)}{F_X(n)}, \quad n\in {\mathbb {N}}_0. \end{aligned}$$

From Theorem 3.1 it follows that if $X\prec Y$ then

$$\begin{aligned} \lim \limits _{n\rightarrow \infty }\frac{O_X(n)}{O_Y(n)}=0. \end{aligned}$$

(ii) *Poisson shock model.* Consider two devices that are subject to shocks occuring randomly in time as events in a Poisson process with constant intensity $\lambda$. Let the probabilities of failure of the devices on the *n*-th shock be respectively $p_X(n)$ and $p_Y(n)$. Then, if $R_X(n)=\sum _{k\ge n}p_X(k)$ and $R_Y(n)=\sum _{k\ge n}p_Y(k),$ the probability that the devices survive until time *t* are given respectively by the formulae (see, e.g., Esary et al. 1973)

$$\begin{aligned} {\overline{H}}_X(t)=\sum \limits _{n=0}^\infty R_X(n)\frac{(\lambda t)^n}{n!}e^{-t} \quad \text {and} \quad {\overline{H}}_Y(t)=\sum \limits _{n=0}^\infty R_Y(n)\frac{(\lambda t)^n}{n!}e^{-t}. \end{aligned}$$

Below we show that if $X\prec Y$ then $\lim \nolimits _{t\rightarrow \infty } {\overline{H}}_X(t)/{\overline{H}}_Y(t)=0.$ By Theorem 3.1, $\lim \nolimits _{n\rightarrow \infty } R_X(n)/R_Y(n)=0,$ and, therefore, for any $\varepsilon >0$ there exists $N\in {\mathbb {N}}$ such that $R_X(n)/R_Y(n)<\varepsilon$ for $n>N$. We then have that

$$\begin{aligned} \frac{{\overline{H}}_X(t)}{{\overline{H}}_Y(t)}&=\frac{\sum \nolimits _{n=0}^N R_X(n)(\lambda t)^n/n!+\sum \nolimits _{n=N+1}^\infty R_X(n)(\lambda t)^n/n!}{\sum \nolimits _{n=0}^N R_Y(n)(\lambda t)^n/n!+\sum \nolimits _{n=N+1}^\infty R_Y(n)(\lambda t)^n/n!}\\&<\frac{\sum \nolimits _{n=0}^N R_X(n)(\lambda t)^n/n!+\sum \nolimits _{n=N+1}^\infty \varepsilon R_Y(n)(\lambda t)^n/n!}{\sum \nolimits _{n=0}^N R_Y(n)(\lambda t)^n/n!+\sum \nolimits _{n=N+1}^\infty R_Y(n)(\lambda t)^n/n!}\\&<\frac{\sum \nolimits _{n=0}^N R_X(n)(\lambda t)^n/n!}{\sum \nolimits _{n=N+1}^\infty R_Y(n)(\lambda t)^n/n!}+\varepsilon . \end{aligned}$$

As $t\rightarrow \infty ,$ the first summand of the last expression goes to 0. Hence, there exists $T>0$ such that ${\overline{H}}_X(t)/{\overline{H}}_Y(t)<2\varepsilon$ for $t>T,$ which means that $\lim \nolimits _{t\rightarrow \infty } {\overline{H}}_X(t)/{\overline{H}}_Y(t)=0.$

(iii) *Sampling with visibility bias*. Recall that formula (15), which defines the order $\prec ,$ was initially motivated by the factorization of rational functions that generate discrete distributions. In the following example we show how the same structural connection between discrete distributions can arise in practice in a completely different setting such as sampling with visibility bias to estimate wildlife abundance.

One of the most practical ways of estimating wildlife abundance are aerial surveys. However, when conducting such surveys, the *visibility bias* caused by various factors, such as snow cover, dense vegetation and observer fatigue, need to be taken into account. There are various techniques proposed in the literature that tackle the problem of population estimation in the face of visibility bias. For example, the quadrat sampling method proposed in Cook and Martin (1974) is based on the exploration of a collection of quadrats within the area of interest. It is then assumed that the number of groups within those quadrats and the size of each group are independently distributed and that each animal is observed with probability *p*. We say that the group of *n* animals is observed if all its members are observed. Then a group of size *n* is observed with probability $p^n$. Now let the random variables *X* and *Y* represent, respectively, the established and the true number of animals in a group. Then we have that

$$\begin{aligned} p_X(n)=cp_Y(n)p^n=\frac{c}{1-p}p_Y(n)p^n(1-p), \end{aligned}$$

where $c=(\sum _{k=1}^\infty p_Y(k)p^k)^{-1}.$ Thus, the above formula is identical to (15) with *Z* following a Geometric distribution with parameter $1-p$.

## Conclusion

We factorize rational functions that recursively generate probability mass functions to obtain a factorization of those probability mass functions. In case the denominator of the generating rational function is a monomial, we show that the probability mass function can be factorized into a product of 4 different types of mass functions, 2 of which correspond to the Poisson and the Negative Binomial distrbutions. This factorization leads to a definition of a stochastic order $\preceq$ on the space $\Omega$ of discrete distributions that are supported on ${\mathbb {N}}_0$. We show that $\{\Omega , \preceq \}$ is a partially ordered set and present its main properties. Remarkably, $\prec$ is not only closed under the operations of addition and multiplication, but is also invariant under relabelings of discrete distributions. In many setups, $\prec$ is also closed under the operation of compounding distributions. The relation $X\prec Y$ also implies a strong relation $\lim \nolimits _{n\rightarrow +\infty }R_X(n)/R_Y(n)=0$ between the survival functions of *X* and *Y*. Comparison of some common distributions with respect to $\prec$ allows to generate new families of distributions that satisfy various recurrence relations. For some of those families, when their members play the role of counting distributions, recursive formulae for the probabilities of corresponding compound distributions are derived. Obtained results have various applications in survival analysis and in aerial census with visibility bias.

## Footnotes

1 $M(1,\eta ,\mu )=\sum \nolimits _{k=0}^\infty \frac{\Gamma (\eta )}{\Gamma (\eta +k)}\mu ^k$ is a confluent hypergeometric function.
